# Septic arthritis caused by *Nocardia asiatica*: a case report and narrative review

**DOI:** 10.1590/S1678-9946202668062

**Published:** 2026-09-18

**Authors:** Mehmet Yildiz

**Affiliations:** 1 Ministry of Health Karadeniz Ereğli State Hospital, Zonguldak, Turkey

**Keywords:** Nocardiosis, Arthropathy, Musculoskeletal disorders, Mass spectrometry, Host defense

## Abstract

*Nocardia asiatica* is a rare opportunistic pathogen typically associated with pulmonary and central nervous system infections; however, musculoskeletal infections are uncommon. We report a case of septic arthritis caused by *N. asiatica* in a 72-year-old man with a history of chronic obstructive pulmonary disease (COPD) and pneumoconiosis. The patient presented with subacute knee swelling following a recent episode of pneumonia. Gram staining of intraoperative synovial samples revealed branching filamentous organisms, and the isolate was identified as *N. asiatica* by mass spectrometry. Although the patient was transferred to another center during the initial phase of management, follow-up information obtained after discharge confirmed a favorable clinical recovery without recurrence. Our report contributes to the limited literature on the clinical manifestations, organ tropism, and antimicrobial susceptibility patterns associated with *N. asiatica* infection.

## INTRODUCTION

*Nocardia* species are aerobic, Gram-positive, filamentous bacteria that cause opportunistic infections. Nocardiosis frequently affects immunocompromised individuals, particularly those with impaired cell-mediated immunity, including patients with hematologic malignancies, transplant recipients, and those receiving immunosuppressive therapy. Nevertheless, cases have also been reported in patients without evidence of systemic immunosuppression. The lungs are the most frequent site of infection, followed by the central nervous system (CNS) and the skin and soft tissues^1^. *Nocardia asiatica* was first described in 2004^2^ and has rarely been reported. Musculoskeletal infections are rare and typically occur following trauma or in the setting of systemic immunosuppression^3^ ^,^ ^4^. To our knowledge, septic arthritis caused by *N. asiatica* in the absence of systemic immunosuppression has not been previously described. Herein, we report a rare case of septic arthritis caused by *N. asiatica*, highlighting its clinical manifestations, microbiological characteristics, and species-level identification by mass spectrometry.

### Ethics

Written informed consent could not be obtained during hospitalization because the patient was transferred to another center. Follow-up information was subsequently obtained through a structured telephone interview, and all personal data were fully anonymized.

## CASE REPORT

**Figure 1:**
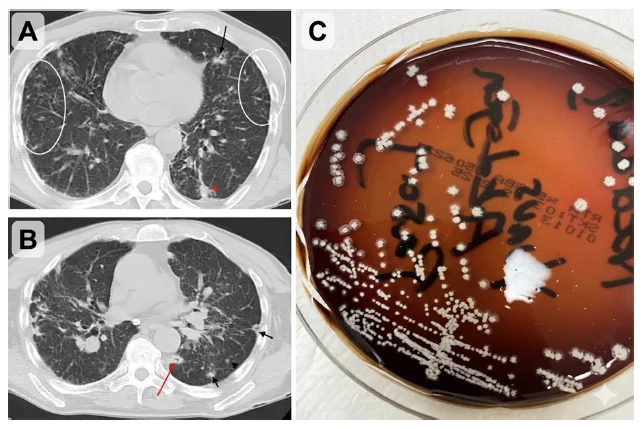
Radiological and microbiological findings of the case. (A) Axial chest computed tomography (CT), pulmonary window, revealing bilateral centrilobular nodules with associated tree-in-bud opacities, predominantly in the peripheral upper lung zones (white circles). An irregular spiculated nodule is present in the lingular segment of the left upper lobe (black arrow), and a small subpleural linear consolidation is observed in the posterior basal segment of the left lower lobe (red arrow); (B) Axial chest CT, pulmonary window, revealing a thick-walled cavitary lesion in the left lower lobe (red arrow), along with several irregular subpleural nodules (black arrows) and a subpleural ground-glass nodule in the left lower lobe (black arrowhead); (C) Colonies of *Nocardia asiatica* on 5% sheep blood agar, showing a dry, chalky-to-powdery, rough, irregularly folded, and adherent morphology with white-to-cream coloration and no distinct odor.

A 72-year-old man with COPD and pneumoconiosis associated with 22 years of quarry work presented to the Emergency Department of Karadeniz Ereğli State Hospital, Zonguldak, Türkiye, with a one-week history of dyspnea. He had no history of malignancy, organ transplantation, or long-term immunosuppressive therapy. However, he reported intermittent, short-term inhaled corticosteroid use for COPD. On admission, the patient was afebrile but tachypneic. Laboratory evaluation revealed significantly elevated inflammatory markers, including a C-reactive protein (CRP) level of 266 mg/L and leukocytosis (Table 1). Chest computed tomography (CT) revealed bilateral multifocal pulmonary abnormalities, including cavitary lesions, centrilobular nodules with tree-in-bud pattern, and patchy ground-glass opacities, consistent with a multifocal necrotizing infectious process (Figure 1A and 1B). Sputum cultures were obtained, and empirical therapy with ceftriaxone plus moxifloxacin was initiated for community-acquired pneumonia. No growth was detected in sputum cultures. No additional respiratory specimens were collected for mycobacterial, fungal, or nocardial investigations during this admission. Following clinical improvement after six days of antimicrobial therapy, the patient was discharged.

**Table 1:** Clinical laboratory findings and antimicrobial susceptibility profile of the *Nocardia asiatica* isolate.

Parameter	Result	Reference range	Antimicrobial agent	MIC value (mg/L)	Susceptibility profile
C-reactive protein (CRP) (mg/L)	266	0-5	Trimethoprim-Sulfamethoxazole	0.125	Susceptible
White Blood Cell (WBC) (× 10³ /µL)	13.4	3.9-10.8	Linezolid	0.750	Susceptible
Neutrophil count (× 10³ /µL)	10.33	1.9-7.5	Doxycycline	1.000	Susceptible
Hemoglobin (g/dL)	10.5	13.5-17.5	Meropenem	1.500	Susceptible
Hematocrit (%)	33.0	39-50	Rifampin	> 32.000	Resistant
Creatinine (mg/dL)	1.25	0.7-1.2	Ciprofloxacin	> 32.000	Resistant
eGFR (mL/min)	54.8	> 90			
Albumin (g/dL)	3.4	3.5-5.2			
Sodium (mmol/L)	133	136-145 /			
Potassium (mmol/L)	5.2	3.5-5.1			
AST (U/L)	42	< 40			
ALT (U/L)	53	< 41			

MIC = minimum inhibitory concentration; Interpretations are based on CLSI M24 criteria as specific EUCAST breakpoints for Nocardia species are currently unavailable.

Two weeks after discharge, the patient presented with progressive pain and swelling of the left knee. Clinical and radiological findings were consistent with septic arthritis, and urgent surgical debridement was performed. Aerobic blood cultures obtained at the second admission yielded no growth. Intraoperative synovial samples were collected, and empirical therapy with ampicillin-sulbactam and vancomycin was initiated. Gram staining revealed branching Gram-positive filamentous organisms suggestive of *Nocardia* spp., prompting the addition of trimethoprim-sulfamethoxazole (TMP-SMX) to the regimen. The isolate was cultured under aerobic conditions at 35-37 °C for 48-72 h (Figure 1C). It was identified as *N. asiatica* by matrix-assisted laser desorption/ionization time-of-flight mass spectrometry (MALDI-TOF MS) (Bruker Microflex system; Bruker Daltonics, Bremen, Germany) according to the manufacturer’s library-based identification system and a validated reference database^5^. Given the risk of hematogenous dissemination, contrast-enhanced cranial magnetic resonance imaging (MRI) was performed and showed no evidence of CNS infection. Antimicrobial susceptibility testing was conducted using the gradient E-test method, and results were interpreted according to the Clinical and Laboratory Standards Institute (CLSI M24) criteria. The isolate was susceptible to TMP-SMX, linezolid, and doxycycline but resistant to ciprofloxacin and rifampin (Table 1). Consequently, therapy was narrowed to imipenem plus TMP-SMX, which was administered for one week. Before completion of the planned treatment, the patient was transferred to another tertiary care center at his request. Therefore, long-term follow-up data were initially unavailable. However, additional information was obtained through a telephone interview approximately nine months after discharge. According to the patient, antimicrobial therapy was continued with oral amoxicillin-clavulanate for approximately four weeks, followed by TMP-SMX for one week. No additional surgical intervention was required after the initial debridement, and the patient reported complete clinical recovery without recurrence or functional limitation.

### Literature review

A PubMed search of the English-language literature was performed using the term ‘*Nocardia asiatica* .’ A total of 25 cases, including the present case, were identified and considered eligible for clinical analysis. The distribution of organ involvement among these cases is summarized in Table 2 ^3^ ^,^ ^4^ ^,^ ^6^ ^-^ ^15^. Pulmonary infection (28.0%), disseminated disease (24.0%), and CNS infection (20.0%) were the most frequent manifestations. Musculoskeletal involvement was observed in three of 25 cases (12.0%).

**Table 2:** Clinical distribution and antimicrobial susceptibility patterns in reported *Nocardia asiatica* cases.

Clinical presentation	n	%	Antimicrobial agent	Susceptible (n/N)	Susceptibility rate (%)
Pulmonary	7	28.0%	Linezolid	9/9	100%
Disseminated	6	24.0%	Amikacin	9/9	100%
Central Nervous System (CNS)	5	20.0%	Ceftriaxone	9/9	100%
Skin and Soft Tissue (SSTI)	3	12.0%	Imipenem	8/8	100%
Osteoarticular	3	12.0%	Trimethoprim-Sulfamethoxazole	11/12*	91.7%
Cardiac	1	4.0%	Amoxicillin-Clavulanate	2/6	33.3%
Total	25	100%	Fluoroquinolones (Ciprofloxacin or Moxifloxacin)	2/7	28.6%
			Macrolides (Clarithromycin)	2/7	28.6%

*The only resistant isolate was reported using a non-standard tube dilution method.

## DISCUSSION

*Nocardia* species, historically classified within the *Nocardia asteroides* complex, have undergone significant taxonomic reclassification with the advent of molecular diagnostic techniques*. N. asiatica*, first described in 2004, remains an infrequently reported pathogen^2^. To our knowledge, this is the first reported case of septic arthritis caused by *N. asiatica* ^1^ ^,^ ^16^. 

Musculoskeletal infections caused by *N. asiatica* have rarely been reported. In 2013, Leitner *et al*.^3^ described a case of post-traumatic olecranon bursitis in an immunocompetent patient. More recently, Suarez *et al*.^4^ reported pelvic osteomyelitis in an immunocompromised patient, which was presumed to have resulted from hematogenous dissemination. Including the present case, only three cases of musculoskeletal infection caused by *N. asiatica* have been reported to date. In our review, musculoskeletal infections accounted for three of the 25 reported cases (12.0%), a proportion higher than the 1%-2% reported in larger nocardiosis series^1^. However, given the limited number of published cases, no definitive conclusions can be drawn regarding tissue tropism, and these findings should be interpreted cautiously.

When isolated organ involvement is considered, pulmonary and CNS infections are the most frequently reported manifestations of *N. asiatica*. This distribution is consistent with previous reports^6^ ^,^ ^10^. However, when all clinical manifestations are considered, disseminated disease emerges as the second most common presentation. Definitions of disseminated disease have varied across studies. In the review by Hayashi *et al*.^10^, only one case was classified as disseminated, largely because a standardized definition was lacking. In contrast, using a definition based on the involvement of two or more noncontiguous organ systems, Banerjee *et al*.^6^ identified dissemination in four of 12 cases (33.3%). Applying the same definition, we identified disseminated disease in six of 25 cases (24.0%). Overall, these findings suggest that systemic dissemination may occur in *N. asiatica* infections at frequencies comparable to those reported in larger nocardiosis series^1^.

According to the review by Banerjee *et al*.^6^, the CNS is the most frequently involved secondary site in disseminated nocardiosis. CNS infections encompass a broad clinical spectrum, ranging from incidental radiologic findings to severe neurological manifestations, including brain abscesses. Therefore, cranial MRI has been recommended even in the absence of neurological symptoms. In our patient, cranial MRI was performed despite a normal neurological examination and revealed no abnormalities.

In this case, the lung was considered the most likely primary site of infection, with subsequent hematogenous spread to the knee joint. Although this sequence could not be microbiologically confirmed because respiratory samples were unavailable, the clinical course supports this hypothesis. The patient had previously received ceftriaxone for pneumonia and showed subsequent clinical improvement. Given the high susceptibility of *N. asiatica* to ceftriaxone (Table 2), this treatment may have provided partial antimicrobial activity without achieving complete eradication, thereby enabling the infection to persist and subsequently disseminate hematogenously. Although blood cultures obtained during the second admission remained negative, *Nocardia* species are infrequently recovered from blood cultures, even in cases of disseminated disease^17^. Taken together, the temporal relationship between the initial pulmonary infection and the subsequent septic arthritis supports hematogenous dissemination from a primary pulmonary focus rather than primary joint infection.

Host factors play a central role in the development of nocardiosis. Patients receiving systemic corticosteroids, chemotherapy for solid organ or hematologic malignancies, or immunosuppressive therapy for rheumatologic diseases, as well as organ transplant recipients and individuals with HIV infection, are traditionally considered immunocompromised hosts. Within this traditional framework, our patient would have been considered immunocompetent^1^. However, advanced age, mild hypoalbuminemia, COPD, and pneumoconiosis may impair local pulmonary defense mechanisms and contribute to immune dysfunction^18^ ^,^ ^19^. Therefore, the patient may be more appropriately characterized as having impaired host defenses rather than being truly immunocompetent. This observation underscores that risk assessment for nocardiosis should extend beyond classical systemic immunosuppression to include local and functional defects in host defense.

The diagnosis of nocardiosis is often delayed because of the slow-growing nature of the organism and its nonspecific clinical presentation. Conventional phenotypic methods, including biochemical testing and susceptibility-based profiling, are time-consuming and may not reliably provide species-level identification. Molecular techniques, such as 16S rRNA gene sequencing and real-time PCR targeting specific genes, improve diagnostic accuracy but are not routinely available in many clinical microbiology laboratories. Metagenomic next-generation sequencing has emerged as a promising diagnostic tool because of its high sensitivity and rapid turnaround time, although its routine use remains limited by cost and accessibility^13^. In our case, molecular confirmation could not be performed because of the lack of in-house testing capabilities. In this setting, early Gram stain findings, together with mass spectrometry, were critical for a timely diagnosis. Although the accuracy of mass spectrometry depends on database coverage and closely related species may exhibit overlapping spectral profiles, species-level identification remains clinically important because antimicrobial susceptibility patterns vary substantially among *Nocardia* species^1^.

Antimicrobial susceptibility data were available for 12 reported isolates. Most isolates were susceptible to linezolid, amikacin, and ceftriaxone, and susceptibility to imipenem was consistently observed. TMP-SMX also remained active against most isolates, whereas fluoroquinolones and macrolides exhibited more variable susceptibility profiles. Consistent with previous reports, our isolate was susceptible to TMP-SMX, linezolid, and doxycycline but resistant to ciprofloxacin and rifampin.

### Limitations

This case highlights a rare presentation of *N. asiatica* septic arthritis in a patient without evidence of systemic immunosuppression, with the diagnosis confirmed by mass spectrometry. However, complementary molecular methods, such as real-time PCR or gene sequencing, could not be performed due to unavailability at our laboratory and might have provided additional confirmatory value. As a single case report, causal inference is limited. Additionally, the literature review was non-systematic and may be subject to selection bias. Although information regarding the final antimicrobial regimen and long-term clinical outcome was obtained, follow-up data were collected through a telephone interview rather than direct clinical evaluation. Therefore, the assessment of treatment response, functional status, and radiological outcomes was limited.

## CONCLUSION

*N. asiatica* is a rare but clinically important pathogen that may cause disseminated or atypical infections, including septic arthritis, even in patients without evidence of systemic immunosuppression. Clinicians should consider nocardial infection in cases of subacute septic arthritis or when routine microbiological investigations are negative, particularly in patients with chronic lung disease or evidence of systemic involvement. Early species-level identification and antimicrobial susceptibility testing are essential for guiding appropriate therapy and improving diagnostic accuracy. Despite advances in microbiological techniques, blood cultures may remain negative in disseminated nocardiosis, potentially delaying diagnosis. This case underscores the importance of considering *Nocardia* species in atypical musculoskeletal infections and highlights the role of rapid diagnostic methods, such as mass spectrometry, in facilitating timely diagnosis and targeted treatment.

## Data Availability

The author will not make additional data available due to ethical restrictions related to patient confidentiality.
